# Nimble vs. torpid responders to hydration pulse duration among soil microbes

**DOI:** 10.1038/s42003-024-06141-5

**Published:** 2024-04-12

**Authors:** Patrick Kut, Ferran Garcia-Pichel

**Affiliations:** Center for Fundamental and Applied Microbiomics and School of Life Sciences, Arizona State University, Tempe, AZ USA

**Keywords:** Microbial ecology, Soil microbiology

## Abstract

Environmental parameters vary in time, and variability is inherent in soils, where microbial activity follows precipitation pulses. The expanded pulse-reserve paradigm (EPRP) contends that arid soil microorganisms have adaptively diversified in response to pulse regimes differing in frequency and duration. To test this, we incubate Chihuahuan Desert soil microbiomes under separate treatments in which 60 h of hydration was reached with pulses of different pulse duration (PD), punctuated by intervening periods of desiccation. Using 16S rRNA gene amplicon data, we measure treatment effects on microbiome net growth, growth efficiency, diversity, and species composition, tracking the fate of 370 phylotypes (23% of those detected). Consistent with predictions, microbial diversity is a direct, saturating function of PD. Increasingly larger shifts in community composition are detected with decreasing PD, as specialist phylotypes become more prominent. One in five phylotypes whose fate was tracked responds consistently to PD, some preferring short pulses (nimble responders; NIRs) and some longer pulses (torpid responders; TORs). For pulses shorter than a day, microbiome growth efficiency is an inverse function of PD, as predicted. We conclude that PD in pulsed soil environments constitutes a major driver of microbial community assembly and function, largely consistent with the EPRP predictions.

## Abstract (web summary)

Theory predicts that bacteria living under pulsed growth regimes adapt to pulse duration by adopting different strategies ranging from nimble to torpid responders. Physiological responses to pulse duration of soil bacteria fit the predictions.

## Introduction

Organisms must acclimate to environmental parameters that typically fluctuate, rather than being constant^1–3^. The consequences of environmental fluctuations may be rather mild, requiring only small physiological responses to cope. For example, episodes of elevated temperature may be dealt with satisfactorily through the deployment of heat-shock proteins to ensure the correct folding of proteins under the new conditions^4^. But fluctuations can also exert severe impacts on physiology, requiring a switch between fundamentally different physiological states characterized by different biochemical and regulatory blueprints. The purple sulfur bacterium *Thiocapsa roseopersicina*, for example, must toggle quickly and recurrently between aerobic chemolithotrophy and anaerobic photolithotrophy to match the diel chemical shifts in coastal sulfidic sediments^5^, requiring extensive biochemical reorganization. Perhaps the most extreme form of fluctuation crosses environmental thresholds so harsh as to impinge on an organism’s overall metabolic activity: full desiccation or extreme cold come to mind. In ecology, when environmental changes are recurrent, intense, and involve a parameter necessary for biological activity, one speaks of “activity pulses”, and of “pulsed ecosystems”, of which polar^6^ or arid environments^7^ are quintessential examples. In pulses involving desiccation, the physiological burden to adaptation is compounded by direct cellular damage to the cell membrane^8^, nucleic acids and proteins^9^. While a wealth of examples exists showing evolutionary adaptations leading to the development of specialist “extremophiles”^10–12^, adaptations that provide fitness to organisms specifically under pulsed conditions are much less obvious. It is even unclear if organisms exist that are evolutionarily specialized to a pulsed existence or to specific types of pulse regimes. Consistent with this notion, however, experiments with microbial communities in biocrusts have demonstrated that a decrease in precipitation pulse size, but maintaining constant total rainfall, results in major relative increases for some cyanobacteria over others^13^, and, similarly, addition of small precipitation pulses gives a field advantage to cyanobacteria over mosses^14^. In any event, nowhere are such pulses better defined and more patent than in surface soils from dryland ecosystems, where fluctuations between water saturation and complete desiccation faithfully follow pulsed rainfall events^15^. There, microbes must toggle between physiological states geared to support (typically short) pulses of activity when hydrated, and (typically long) periods of quiescence when dry^16,17^. This extremely pulsed regime makes arid top-soil microbiomes prime targets of inquiry in this arena.

Theoretical considerations suggest that organisms in pulsed environments are indeed adapted to pulse variability, not just to the end-member situations within a pulse (i.e., high and low salinity, inactive and desiccated vs active and hydrated, oxic vs. anoxic). In other words, their adaptations are suited to surviving the changes themselves because to transition between appropriate physiological blueprints in a pulse is costly^18–20^ and it takes time. Time is particularly important under regimes where growth-enabling time is at a premium. The long-standing pulse-reserve paradigm (PRP) of arid ecosystem function calls for the central role of organismal reserves gathered during times of plenty to power such transitions effectively^21,22^. Pulse duration (PD) will determine whether enough reserves can be acquired to eventually start a new growth phase. Short pulses may end up costing more resources than can be acquired, leading to organismal demise. According to theory^23^, adaptations to a pulsed existence will fall along a continuum between two end-member strategies. At one end “Nimble Responders (NIRs)” transition swiftly in and out of growth mode by constantly allocating a proportion of resources to reserves, maintaining a constitutive physiological readiness for inter-pulse conditions, and ensuring that metabolic systems are inherently hardy and protected. This comes at the cost of depressing their growth potential during the activity pulse; they become inherently slow growers. At the opposite end, “Torpid Responders (TORs)”, must also allocate resources to reserves to fuel transitions, but they only do so as an activity pulse nears its end. Consequently, TORs fulfill their growth potential during much of the pulse, unconstrained by allocation to reserves, and they grow swiftly during most of it. But their lag times for transition to dormancy and back into growth are long, as reserves are synthesized ad hoc at the end of a pulse. TORs also have comparatively long minimal pulse duration for viability. NIRs can be understood as conservative investors, focused on certainty of returns that are moderate but frequent, while TORs act like risky investors whose strategy relies on high returns that occur rarely. Specific organisms will fall within a continuum between the extremes exemplified in the NIR and TOR acronyms.

The NIR-TOR continuum theory, while consistent with available phenomenology and some anecdotal evidence, has yet to be tested directly. We sought to conduct an experimental, quantitative test of some of its predictions. Specifically, we asked if one could indeed find evidence for physiological diversification of soil bacteria along the NIR-TOR continuum, and if this would translate into a community’s overall growth capacity. To do this, we used (heterotrophic) dryland soil microbiomes from the aphotic zone of biological soil crusts and subjected them to defined pulsed hydration incubation regimes with a diverse carbon source. We then determined the changes that ensued in basic microbial diversity and growth parameters, as well as the fates of the populations of hundreds of phylotypes as a function of pulse duration to show that, indeed, pulse duration strongly influences structural and functional properties of microbial communities on soil.

## Materials and methods

### Source soil and preparation

**Fig. 1:**
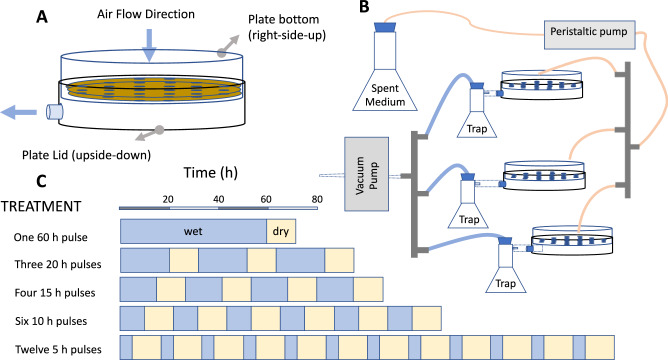
Experimental assembly and design used for incubations of biocrust soil under controlled hydration pulse regimes. **A** Soil incubation chambers constructed from Petri dishes. **B** Connection of chambers to vacuum and medium sources for desiccation and wetting. **C** Pulse regime treatment schedule, with wetting (blue) and dry (yellow) periods. Blue periods always add to 60 h, dry times are invariably 12 h.

As a main experimental material, dry biocrust soil was collected in the Jornada Long Term Experimental Range near Las Cruces, New Mexico (32°34'06.9“N, 106°45'29.4“W). The biocrust were of the light-crust type and dominated by the cyanobacterium *Microcoleus vaginatus*^24^. The 2-mm thin phototrophic layer of the biocrust was peeled off the surface by hand, and the soil below was collected down to a depth of 2 cm. The removal of phototrophs was done for the sake of experimental simplicity since biocrust phototrophs have doubling times in the order of 1–15 days ^25^, which would have imposed a far longer experiment, and also to avoid cascading effects since heterotrophs would be dependent on phototrophic exudates. The soil was sifted through a 4.75 mm sieve to remove or break up larger particles, transported and stored in a closed bucket at room temperature until experiments were started, then homogenized by shaking and mixing by hand. Plates for incubations were constructed as shown in Fig. 1, provided with sterile paper filters at the bottom and filled with 40 g of homogenized soil to a soil depth of 3–4 mm. All tubing, manifolds, and plates were either autoclaved or sterilized in ethanol and dried under UV in an engaged laminar flow hood. The incubation plates were assembled using one Petri plate lid and one bottom, the lid rim glued to the bottom side as shown in Fig. 1A. The bottom section had 30 interspaced holes to allow the exit of the soil solution when under vacuum and the lid had a side hook-up for a vacuum line.

### Treatments

Five treatments were designed to isolate pulse duration as the independent variable. They included variations of the number and duration of pulses so that all had a total active (hydrated) time of 60 h. Treatments experienced 12 h long dry periods between pulses. The treatments were: never wet (control), 12 five-hour long wet pulses (5 h), six ten-hour long wet pulses (10 h), four 15-h long wet pulses (15 h), three 20-h wet pulse (20 h), and one 60-h wet pulse (60 h). A graphic schedule of treatments is in Fig. 1. Desiccation to 5% water content in this set-up was enhanced by vacuum and was attained within 4.3 h after the end of the wet period as measured by a conductivity-based water content miniprobe (UP Umweltanalytische Produkte GmbH, Ibbenbüren, Germany) in situ (Fig. S1). Plates were kept in an engaged laminar flow hood through the incubation to prevent external contamination. To prepare an appropriate medium for wetting the soil, the cyanobacterium *Microcoleus vaginatus* strain PCC9802 was grown in 50% strength BG-11 medium for two weeks at room temperature in flasks with vented-caps under a 12 h photoperiod^26^. The spent medium was then filter-sterilized by passage through a 0.2 µm pore diameter filter and used as a close-to-natural source of diverse C compounds^27^ for the communityt. At the end of each prescribed wet period, the soil solution was actively suctioned under mild vacuum (−0.22 bar) for one hour, then kept dry for 11 h. Soil moisture dropped below 5% water content, within 4.3 h, which is not an unusually fast desiccation speed compared to those experienced in crusts during summer conditions, remaining at levels too low for microbial activity^28,29^ until the next wetting (Fig. S1). These regimes resulted in significant and sustained soil respiration at each wetting, but the soils never got anoxic, even at depth (Fig. S1), as measured directly in situ using a O_2_-measuring microoptode with a 50 µm tip diameter, connected to a Fire-Sting O_2_ oxygen meter, both from Pyroscience Gmbh (Germany). After the final drying cycle, dishes were stored wrapped in parafilm and covered in tin foil at 4 °C. Once all treatments were completed samples were promptly processed.

### DNA extraction and molecular analyses

Three 0.25 g samples of soil were taken from each plate to extract community DNA with Qiagen DNeasy PowerSoil Pro Kit (Qiagen Inc. Germantown, MD, USA) used according to the manufacturer’s instructions. DNA concentrations in the extracts were determined initially via Qubit (Qubit 3 Fluorometer Invitrogen by ThermoFisher Scientific, Waltham, Massachusetts, USA). Guided by these data, extracts were diluted for qPCR to quantify the content of 16S rRNA gene copies and amplified using universal primers 338F (5′-ACTCCTACGGGAGGCAGCAG-3′) and 518R (5′-ATTACCGCGGCTGCTGG-3′)^30^. The PCR reaction was performed in triplicate alongside negative controls of molecular grade water using SYBR Green FastMix ROX Quantabio (Beverly, Massachusetts, USA) under the following conditions: an initial denaturation phase (2 min at 98 °C), followed by 40 cycles of denaturation at 95 °C for 10 s and annealing at 55 °C for 30 s, followed by the melting curve acquisition (increasing temperature from 55 to 95 °C at 0.5 °C s^-1^ speed)^31^. This qPCR produced a standard curve with an R^2^ of 0.998. The standard curve was then used to calculate the number of copies of the 16S rRNA gene per mg of soil.

Additionally, 20 µL of the same extract as used for qPCR for each sample was used for 16S rRNA gene sequencing with MiSeq sequencing (Illumina NGS) with the 515F/806R primers which target the V4 region of the 16S rRNA gene^32^.

### DOC additions, quantification and balances

15 mL of the spent *Microcoleus* medium were initially added per wetting event to all treatments to achieve soil saturation. This corresponds to a rain event of 2.3 mm (15 mL over a surface 64 cm^2^ surface), which is within the low end of rain event size at the site of origin (Fig. S2). To maintain soil hydration throughout the full duration of the wetting pulse, an additional 3 mL was added every five hours thereafter to bring the soil to saturation. The soil was wet to saturation as dryland soils are commonly saturated and experience runoff following rainfall. Total DOC added per pulse was computed from total wetting volume and the DOC concentrations of the spent medium (39 mg L^-1^), and was set to match natural steady state levels of biocrust in the soil solution^33–35^, the totals added varying between 0.9 and 3.0 mg per plate, so as to balance consumption in longer incubations. Net DOC accumulation in the soil after incubation was determined by subtraction of the DOC content of untreated control soils (0.93 mg per plate or 0.02 mg/g) from the end-point DOC content in each plate. The additions thus maintained the DOC concentrations well within the very low DOC (oligotrophic realm) natural for these soils. Dissolved organic carbon extraction from soils was performed on 20 g (50%) of each plate, following ref. ^36^. All DOC determinations were processed by Arizona State University Metals Environmental and Terrestrial Analytical Laboratory for total carbon and dissolved organic carbon (DOC) quantification by an elemental analyzer.

### Bioinformatic analyses

16S rRNA gene sequences obtained were demultiplexed and quality controlled using the DADA2 plugin of Qiime2 2022.8 refs. ^37,38^. Raw sequences were then trimmed for quality control, which created a feature table of unique sequences (amplicon sequence variants, ASV) along with their frequency of occurrence. Singletons were disregarded from this table. The sample with the least reads had 11939, and that with the most had 28484 (Fig. S3). To ensure equal sampling effort, all samples were trimmed randomly to include a sampling depth of 11939. Alpha rarefaction analysis showed that this was sufficient to reach saturation of diversity assessment in all samples (Fig. S3). ASV were initially automatically classified using Greengenes 13.8 ref. ^39^. and any Archaeal reads discarded, but more detailed taxonomic assignments were carried out through BLAST for any ASV of interest, given the low resolution and high level of errors in this database^40^. BLAST assignments were conducted using the 16S gene sequence and the strain with the highest percent identity was selected with all of the sequences which underwent BLAST having 100% similarity with a sequence in the NCBI database. Disagreements between the Qiime2, Greengenes 13.8 assignment and BLAST were handled by selecting the taxonomic classification from BLAST as Greengenes has been demonstrated to have a high error rate^41^. When a specific strain could not be selected as multiple different sequences shared 100% similarity it is noted using a “/”. Qiime2 2022.8 was used for all diversity metrics including Shannon’s Diversity, Chao1, Weighted UniFrac, Unweighted UniFrac, Jaccard’s index, and Bray-Curtis index. Beta-diversity was assessed using principal coordinates analysis (PCoA) in Qiime2. From Qiime2, coordinates were extracted and plotted in R so that 95% confidence ellipses could be calculated with the R vegan package^42^. Differential abundance analyses were also done in R using Hellinger to normalize the feature table created above. ASVs which were differentially abundant between the 5-h and 60-h pulse regimes were determined using the DeSeq2 method^43^ in the R BiocManager package.

### Determination of nimble responder (NIR) vs. torpid responder (TOR) character

Linear regressions of log base 2 abundance as a function of treatment duration (excluding controls) were conducted in R on data from any ASVs detected in at least six different samples. This culled ASVs to 370 out of an original 1639 detected. The slopes of such regressions are a proxy for the NIR vs. TOR character of an ASV, where NIRs would show consistently increasing abundance with shorter pulse and vice-versa. To do this properly absolute counts are needed, but absolute counts are lost on sequencing because of the intervening PCR reaction. To restore an absolute measure of abundance, we multiplied each ASV’s relative abundance by the total number of 16S rRNA gene copy counts per unit weight of soil determined by qPCR on aliquots of the same extract that was sequenced.^44^. Because this is an indirect measure, we refer to it as Restored Absolute Abundance (REA). A log_2_ transformation of REA values was then conducted to yield units that reflect apparent number of doublings. To assess the statistical significance of regressions, we adjusted p values using the Benjamini-Hochberg correction to account for the multiplicity of statistical analyses conducted.

### Statistics and reproducibility

All statistical analyses were performed in R statistical software version 4.1.2 (2021-11-01) refs. ^42,45–48^. Levene’s Test was used to test for equal variance and Shapiro-Wilk Test for normality. After verifying that assumptions were met, the data was analyzed by Analysis of Variance (ANOVA). Tukey’s honest significant difference was used as a post-hoc test for pairwise comparisons. The experimental design was based on the detection of trends in measured or derives parameters (reponse variables) as a function of increased severity of treatments, and as such it contains an inherent measure of reproducibility with an n of 5 against controls.

### Reporting summary

Further information on research design is available in the Nature Portfolio Reporting Summary linked to this article.

## Results

### Pulse regime effects on microbiome diversity

**Fig. 2:**
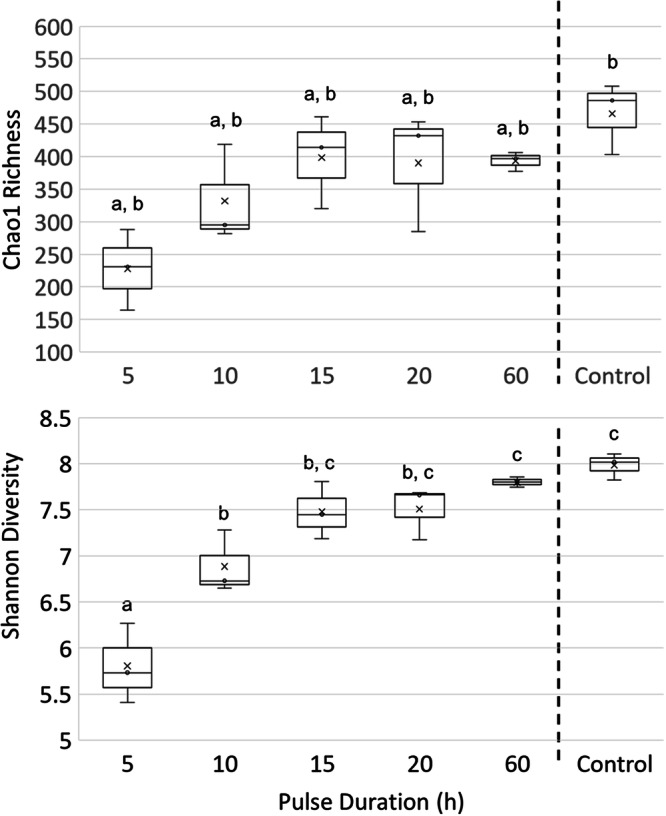
Effects of incubation under varying hydration pulse regimes on diversity of the soil microbial communities based on 16S rRNA gene sequence variants (ASVs). Estimators of Richness (Chao1 Richness) and Diversity (Shannon Diversity) showed congruent trends of increase with increasing pulse duration. Data shown as box-and-whisker plots with *n* = 3, where x are means, lines are medians and bars are two standard deviations. Different letters above boxes indicate significant differences (*p* < 0.05) in post-hoc tests. Explicit log fits of the trends with pulse duration are in Fig. S4.

We gauged microbial community diversity in the soil under different pulse regimes using both Chao1 and Shannon diversity indices applied to the 16S rRNA gene data (compositional) at the maximal phylogenetic resolution possible (ASVs level; Fig. 2; detailed counts can be found in Supplementary Data 1). Data binned by treatment had equal variances for both indices, and data for both indices were normally distributed according to, respectively, Levene’s and Shapiro-Wilk tests. Therefore, ANOVA was an appropriate statistical test. It showed significant differences with treatment for both Chao1 Richness (df = 12, *F* = 4.2, *p* = 0.019) and Shannon Diversity (df = 12, *F* = 25.0, *p* = 6 × 10^−6^). We found a depression of community diversity with decreasing pulse duration, the 5 h treatment being only half as rich as the control in the case of Chao1 and with a similar decline when considering Shannon Diversity. These declining trends in diversity and richness with shorter pulse duration were not only significant but fit a logarithmic (saturating) function of PD best (R^2^ = 0.35 with *p* = 0.02 for Chao1 and R^2^ = 0.75 with *p* = 3 × 10^−5^ for Shannon; Fig. S4), apparent Chao richness and Shannon diversity loses thus intensifying in the short pulse range.

### Pulse regime effects on community composition

**Fig. 3:**
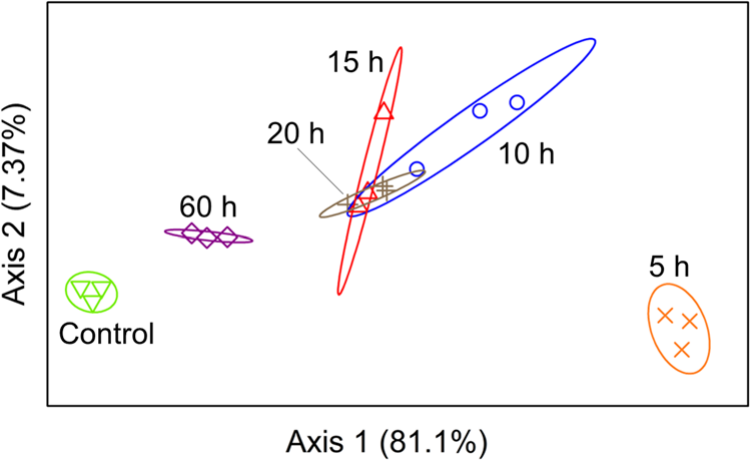
PCoA analyses of similarity in microbial communities as a function of pulse duration treatment based on Weighted UniFrac similarity. Ellipses, color coded to match the treatments, indicate the 95% confidence area for the respective treatment.

The treatments caused clear shifts in microbial community composition, while replicates within treatments remained rather self-similar. This we could demonstrate quantitatively using a PCoA ordination based on Weighted UniFrac distances (Fig. 3), which measures community similarity based on both relative abundance of its members and their phylogenetic distance^49^. This analysis explained 88% of the variability in a two-axis space. Communities became increasingly more differentiated from the starting (Control) community the shorter the pulsed duration imposed. PCoAs based on other similarity algorithms are shown in Fig. S5. Bray-Curtis similarity, which separates community by relative abundance only^50^, yielded very similar results to those shown in Fig. 3, albeit with a somewhat reduced explanatory power (64% of variability). However, algorithms based on presence/absence only, with (Unweighted UniFrac) or without phylogenetic distance (Jaccard’s Index)^51^, did not provide good separation. This all implies that differences in relative abundance of particular ASVs, rather than the appearance or disappearance of phylotypes drove the diversification in community composition. ASVs responding consistently to treatments apparently had some level of phylogenetic relatedness, judging from the slightly better separation capacity of Weighted UniFrac over Bray-Curtiss algorithms, although this phylogenetic signal was not very strong.

### Pulse regime effects on growth efficiency

**Fig. 4:**
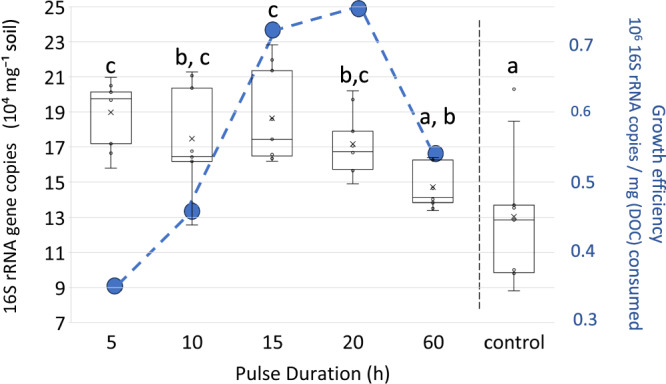
Microbiome size and growth efficiency as a function of pulse duration regime. Left scale, in black: box-and-whisker plots of 16S rRNA gene copy counts (*n* = 9), where crosses are means, lines medians, and error bars two standard deviations. A linear regression of microbiome size vs. pulse duration has a *p* = 9 × 10^−5^ and R² = 0.30 (see Fig. S4). Right scale, in blue: growth efficiency as a function of pulse duration computed from balance sheets in Table 1.

We define the overall size of the microbiome as simply the absolute content of 16S rRNA gene copies in it, and microbiome growth or decline as the change in this parameter. We could detect significant net growth of overall bacterial populations (microbiome) defined in this way under different regimes with respect to the concentration found in the untreated soil (Fig. 4). These data had equal variances and were normally distributed. Therefore, an ANOVA was an appropriate statistical test; it showed differences with treatment to be significant (df = 48, *F* = 7.3, *p* = 4 × 10^−5^). The increases ranged in magnitude from 12% to 45% and were significant in all but the 60-h treatment, according to post-hoc Tukey Honest Significant Difference tests. In fact, there was a trend of decrease in growth with PD, significant by regression analysis (Fig. 4 and S4). Even in the most active treatment, microbiome growth was meager. The microbiome grew much less than a doubling, which corresponds to generation times much longer than those typical for environmental bacteria^52^ and suggests a potential limitation to growth. As shown in the balance sheets of Table 1, the DOC provided was almost completely consumed by resident bacteria at each pulse in all treatments, and the oxygen dynamics in the soil showed a slowing of respiration rates soon after additions of DOC (Fig. S1), both confirming the notion of a C limitation. In fact, the net microbiome size increases paralleled the total DOC consumed (R^2^ = 0.62 with *n* = 5). Interestingly, however, the growth efficiency (microbiome growth per unit DOC used; in blue in Fig. 4, calculations in Table 1) increased with PD until 20 h, to then suffer a moderate decrease again at 60 h. It could be suggested that the growth efficiency decrease with long water-saturated incubations could be due to anoxia (and fermentation) setting in, but direct measurements show that this was not the case (Fig. S1).

**Table 1:** Balances sheets for dissolved organic Carbon (DOC) and microbiome size parameters as a function of treatment

Treatment (pulse duration)	Number of pulses	Dissolved Organic Carbon						Microbiome Size		
		End-point content	Consumed (cumulative)		per	pulse		Increase	Doublings	Growth efficiency (million 16 S copies / mg DOC consumed)
				Provided^a^	Excess^b^	Consumed	Efficiency (%)^c^			
								(cumulative)	(cumulative)	
5 h	12	1.3	6.68	0.59	0.03	0.56	94.75	2.37	0.54	0.35
10 h	6	1.34	3.82	0.70	0.07	0.64	90.31	1.77	0.42	0.46
15 h	4	1.1	3.12	0.82	0.04	0.78	94.83	2.24	0.52	0.72
20 h	3	1.55	2.20	0.94	0.21	0.73	78.01	1.65	0.40	0.75
60 h	1	0.86	1.24	1.17	−0.07	1.24	105.98	0.67	0.17	0.54

All values are per incubated plate and average for all plates.

^a^computed from cumulative volume of spent medium additions at a concentration of 39 mg DOC/L

^b^computed from difference between end-point measurements and average initial (controls) content of 0.93 mg DOC

^c^as % of DOC consumed for DOC provided

### Pulse regime effects on individual phylotypes

**Fig. 5:**
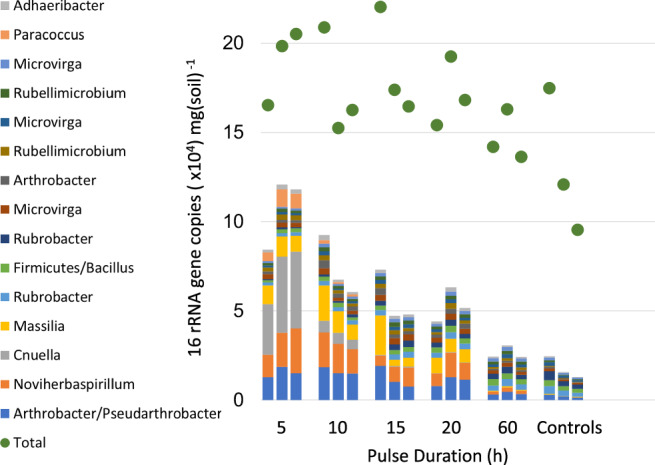
Pulse duration effect on bacterial abundance. Restored absolute abundances of total bacteria/archaea (green dots) and of individual ASVs phylotypes (bar plots, by color as in the legend) as a function of pulse duration treatment. All replicates are shown independently. For clarity, only the 15 most abundant ASVs are shown. The best possible taxonomic assignment for each ASV is given in the legend to the left.

**Fig. 6:**
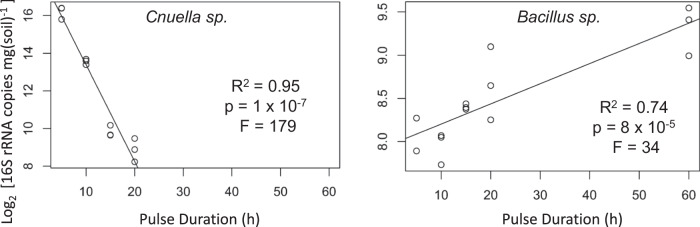
Impact of pulse duration on ASV abundance. Exemplary impacts of pulse duration on restored absolute abundance of particular ASVs according to specific 16S rRNA gene copy counts. Scatter plots with regression analyses for a *Cnuella* ASV (*n* = 12) shows preference for shorter pulses (NIR-type) and an ASV in the genus *Bacillus* (*n* = 9) shows preference for longer pulses (TOR-type). Comprehensive analyses are gathered in Fig. 7 and in Supplementary Data 2.

We obtained absolute abundance of individual ASVs, as opposed of relative abundances used for alpha and beta diversity determinations above, by combining relative ASV abundances from high-throughput sequencing with a determination of total 16S rRNA gene copy counts per g of soil for each treatment and replicate, as customarily done in similar systems^26,53^. A graphical depiction of these data, simplified to showcase just the 15 most common ASVs, is in Fig. 5. The complete set is found in Supplementary Data 1. The fraction of all 16S rRNA gene counts assignable to the most common 15 ASVs increased with shorter pulses, consistent with the decreased diversity we saw in short-pulse treatments (Fig. 4). When comparing the trend of absolute abundances as a function of pulse duration in these most common bacterial ASVs, differential fates (growth vs. demise) of specific bacteria become apparent: some fared better under shorter pulses, while some apparently preferred longer pulses. In order to quantify these responses, we carried out regression analyses of ASV-specific counts (log base 2) as a function of pulse duration. An example of an ASV that did significantly better with shorter pulses is ASV 30970ef (assignable to the genus *Cnuella*) as shown in Fig. 6 (*p* < 0.05, R² = 0.95, df = 10, *F* = 179), while an ASV assignable to the genus *Bacillus* (ASV 97fd540) had a clear preference for longer pulses (*p* < 0.05, R² = 0.74, df = 12, *F* = 34) (Fig. 6). *Cnuella*’s response is what would be expected of a NIR organism in the theoretical framework of Garcia-Pichel and Sala (2022) [ref 23.], competing more successfully for resources under shorter pulses than under longer pulses. The *Bacillus* ASV, on the other hand, did better with longer pulses, which would be typical for TOR-like organisms. We could conduct this kind of systematic analysis on a total of 370 different ASVs. Of those, more than 20% presented either a highly significant (*p* < 0.05; corrected for multiple comparisons) or a significant (0.05 < *p* < 0.1) correlation. The remaining 296 were not significantly responsive to PD length (*p* > 0.1). Of the ASVs with significant regressions, some 60% tended to be TOR-like organisms (i.e., doing better under long pulses, with a positive slope), and 40% showed a NIR-like response (negative slope). NIRs tended to display higher absolute values of the regression slope than TORs, in essence responding more decidedly to variations in PD.

### Who is a NIR and who is a TOR?

**Fig. 7:**
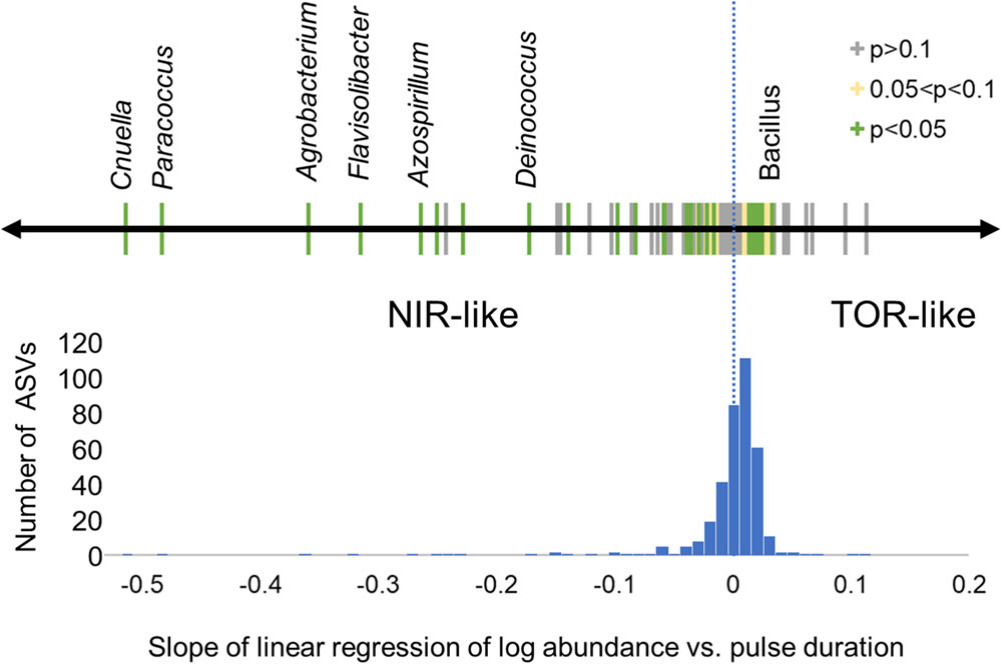
Phylotypes mapped to the NIR-TOR continuum. Placement of ASVs in the NIR-TOR continuum quantified as the slope of regressions like those in Fig. 6. Each vertical line represents a single ASV, color coding denoting the *p* value of its corresponding regression. Some taxonomic assignments are indicated. The histogram below shows the distribution of ASV placements along the slope continuum.

Individual ASV’s with marked NIR or TOR character are placed on a quantitative scale in Fig. 7, based on the slopes of the regression attained. A complete list of ASVs with their taxonomic assignment and their associated regression statistics is in Supplementary Data 3. Even a cursory look at this table or Fig. 7 will show that there is no clear phylogenetic (taxonomic) group of bacteria specialized as either NIR or TOR. For example, ASV’s in the genera *Cnuella*, *Agrobacterium* or *Paracoccus*, belonging to separate Phyla, are among the most clearly NIR phylotypes, and have apparently attained this character convergently. This notion is also supported by analysis of key drivers in community difference between the 5 h and 60 h pulse regimes shown using DESeq differential abundance analysis which demonstrated that 66 ASVs were differentially abundant between the 5 h and 60 h pulses (*p* < 0.05) (Fig. S6). And yet, the phylogenetic distribution of PD preference was not random either. All five *Deinococci* ASVs detected are on the NIR side with negative slopes, and many NIR-like organisms gather conspicuously around the families *Cytophagaceae* and *Chitinophagaceae* (both in the Phylum *Bacteroidetes*), and around the *Oxalobacterales* (*Betaproteobacteria*) and *Rhodobacterales* (*Alphaproteobacteria*). One should caution, however, that all or part of the apparent diversity in some of these closely related ASVs could potentially stem from sequence variability among homologs when strains contain more than one copy of the 16S rRNA gene in their genome. Strongly leaning to the TOR side of the slide we consistently find ASVs in the classes *Chloracidobacteria*, “Acidobacteria-6” and *Solibacteres* (all three in the Phylum *Acidobacteria*), in the Actinobacteria (Classes *Actinobacteria* and *Rubrobacteria*), as well as many in the order *Bacillales* (*Firmicutes*).

It may be worth noting that the list only applies to heterotrophs, which was the target of our experimental design. Chemolithotrophs and photoautotrophs were not considered, even when they can be important members of the biocrust community^15^ and should principally also respond to the constraints of the PRP.

## Discussion

Our work indicates that shortening the duration of activity pulses will decrease bacterial diversity, both in terms of richness and diversity. This is consistent with the progressive decreases in diversity measured in less controlled, but perhaps more natural, settings, where the frequency of desiccation varies naturally across spatial gradients^54^ or was experimentally altered^55^. The implication that a progressively smaller number of bacterial species can cope with a more intensely pulsed growth regime seems thus to be generalizable. Mechanistically, an explanation for such an effect can be sought in the expanded PRP: the increasing physiological burden imposed by frequent transitioning between cellular quiescence and growth states depresses the net potential biomass gain a microbe can inch from a pulse, as a larger proportion of resources and energy must be allocated to transition-powering reserves. Additionally, of course, direct cellular damage and stress caused by frequent desiccation^9,56^ may also contribute to this effect. Few specialists will thus cope. The decreases in growth efficiency measured (Fig. 4) with shortening pulses below 20 h are consistent with this notion. In this regard, pulsed environments of short PD should be considered extreme, in the same way that progressively fewer species characterize environments more extreme in mean values of environmental parameters like temperature, salinity, etc.^57^.

That the untreated control would display the greatest diversity of any sample is also consistent with ecological expectations. It should have been exposed to pulses of varying duration in the natural environment during its recent past, thus allowing for an array of physiological types along the NIR/TOR continuum to time-share opportunities for resource acquisition, resulting in a more diverse assemblage, consistent with the notion that environmental variability generally furthers microbial diversity^3,58^. Interestingly, in the US Southwest, climate change models predict a decrease in the PD of summer rainfall^59^, a trend that is in fact already measurable^60^. Under this climate-change scenario, and judging from our results here, the source soils for our experiments will (and should already) experience loses in microbial biodiversity and functional versatility.

Beyond topsoil microbiomes, microbial communities where adaptations to extreme pulsing can be expected to play significant roles include rain-driven subaerial biofilms, intertidal microbial mats, or intertidal endolithic communities, and the aerobiome as it transitions between dry aerosol and hydrated cloud/fog states. Recurrent desiccation and rehydration pulses are characteristics of all of them.

Our analyses found clear evidence for the physiological diversification of bacteria in dryland soils along a gradient of preference for either short or long PD (NIR vs. TOR), as postulated in the expanded pulse-reserve paradigm (EPRP) of Garcia-Pichel and Sala (2022) [ref. ^23^]. The intensity of the community responses detected in our experiment, which was rather short in total duration, is somewhat surprising and speaks for the strength of PD as an ecological driver. That we could detect significant, consistent responses to PD involving 1 in 5 ASVs for which enough data could be gathered, likely implies that many more ASV could have been detected had the treatments lasted longer, involved larger changes in total microbiome size, or if simply we had used deeper sequencing.

The growth preferences for PD we detected fell roughly on either side of the NIR/TOR continuum middle point defined by our experimental PD range. On the basis of typical bacterial doubling times, it was predicted^23^ that NIR-like microorganism would become prominent during pulses shorter than 24 h while TOR-like organisms would prefer pulses longer than a day. The PD range used in our experiment was designed to reflect inclusively conditions experienced during Summer Monsoon-type rains, in which topsoil may remain wet for hours only, to the duration of typical winter rain events where the topsoil may remain wet for a few days. This implies that the divergence in physiology we found should result in tangible advantages or disadvantages under current natural rainfall pulse regimes. Because a Summer monsoon season in warm deserts may only provide total wet times in the order of our experiment’s duration, it also allows us to predict that rain PD should also be a driver of seasonal population dynamics in the soil microbiome through the NIR/TOR character, in addition to temperature (i.e., refs. ^61,62^), because of the PD differential associated with summer vs. winter rain events. Unfortunately, the absence of seasonal population dynamics data remains currently one of the most conspicuous knowledge gaps in biocrust research^15^.

The phylogenetic distribution of the NIRs and TORs we could detect constituted an interesting aspect of our findings: while neither NIR nor TOR character was exclusive to one or a few taxonomic/phylogenetic clades, evidence from both the optimal explanatory power of ordination approaches that include phylogenetic distance (Fig. 3 vs. Fig. S5), and the recurrent appearance of certain taxa in the lists of NIRs and TORs speak for a moderate degree of heritability. Based on the types of physiological adaptations to desiccation (for example, formation of spores with long periods of germination) some taxa were predicted to fit the TOR pattern: Actinobacteria and Firmicutes^23^. Desiccation-induced spores^63,64^ represent an extreme case of all-out late-pulse allocation to reserves that enables extremely long quiescence periods but requires a relatively long pulse (days) with copious resources to enable a complex process of germination^64,65^, as demonstrated in the *Bacillus* blooms elicited during long wetting pulses in biocrusts^66^. Other complex patterns of response to (a single) wetting pulse^67^ could potentially provide some additional guidance. Our survey at least partly confirmed this prediction, although the experimental patterns we found were far from absolute, containing many an exception.

In our attempt to compare theory and experimental evidence, a limitation must be highlighted: the NIR/TOR theory is based on the differential physiology of organisms in isolation, whereas the experiments carried out here measured responses of microbial communities and their members based not only on their differential physiology, but also on how it plays out in the presence of other members and any ensuing interactions. For example, the theory predicts that, in isolation, both NIRs and TORs will do better with long than in short pulses. It is only under competition for resources (i.e., space, carbon, nutrients) that NIRs will lose ground with longer pulses, at which they are less efficient. Thus, our interpretation that NIR’s populations will decline with longer pulses inherently necessitates that competition be at play, and this is why our experiments were designed to maintain C limitation. By contrast, the NIR/TOR model predicts that TORs will inherently do worse with short pulses because they have longer minimal PD for viability. Perhaps this discrepancy is behind the finding that the typical TORs sensitivity for pulse size (i.e., the absolute slopes in Fig. 7) was more moderate than that of NIRs.

The other predictive limitation of consequence is that the theory includes mechanistic explanations about nimble or torpid responses on the basis of differential management of storage compounds. But our experiment offers no mechanistic information in this regard. Of course, microbes do commonly produce organic and inorganic storage reserves, typically in the form of polymers^68^, the genetic ability to produce abundant reserves correlating in specific microbes with resistance to stress conditions^69^, and demonstrably supporting resuscitation from dormancy^70^. But the databases currently do not allow us to connect directly phylogenetic assignments with reserve physiology for a full trait-based analysis, other than perhaps pointing to the coincidence between spore formation capacity and TOR character discussed above. This will have to await more directed research efforts.

The theoretical expectation for any given microbe was that as more carbon is allocated to powering transitions to quiescence and resuscitation with decreasing PD, less of it would be available for net growth, so that, all other things being equal, growth rates should decrease with PD. And yet we found that net growth of the microbiome was significantly more pronounced with decreasing PD (Fig. 4), which can be apparently paradoxical. However, in our experiments, DOC availability was clearly limiting growth, as practically all DOC provided was consumed in all treatments (Table 1), and respiration never quite consumed all available oxygen (Fig. S1). Because of this, net microbiome growth followed roughly DOC availability, which was unequal among treatments. However, the calculated values of growth efficiency were more in line with theoretical predictions, generally decreasing with shortening PD (Fig. 4), with the notable exception of the 60-h treatment, which had only moderate growth efficiency. Potentially, this long treatment may have suffered from a depression in respiratory efficiency because of more generalized microoxic conditions in the soil, and a lesser availability of high-quality, readily metabolizable DOC (Fig. S1). In any event, the functional properties of the soil microbiome are dependent on PD in a manner consistent with theoretical expectation for pulses around or below a day’s length.

The recently advanced “expanded pulse reserve paradigm” offers a theoretical framework to understand and predict microbiomes in arid lands on the basis of activity pulse regimes driven by water availability, but its predictions had not been subject to direct experimental testing. Here, we provide evidence for some of the paradigm’s main phenomenological predictions, including the existence of specialist microbial types along the NIR-TOR continuum, and the direct relationships between microbial diversity and growth efficiency as a function of pulse duration. No attempts were made to test the central mechanistic prediction of the EPRP that the phenomena observed are based on the adaptive diversification of physiological management of reserves. This missing assignment will likely require more sophisticated metagenomic and metatranscriptomic analyses targeting reserve polymer genes on natural assemblages, or more traditional physiological studies on new TOR and NIR-like isolates. Such isolates have not been obtained either.

### Supplementary information

Supplementary Information

Description of Additional Supplementary Files

Supplementary Data 1–5

Reporting Summary

## Data Availability

All data supporting the findings of this study are available either within the paper or its Supplementary Information. The source data behind the graphs in Figs. 2 and 4 can be found in Supplementary Data 4 and 5 respectively. The sequences used have been deposited into NCBI database with BioProject accession number: PRJNA996965.
