# Supplementary material for: Nimble vs. torpid responders to hydration pulse duration among soil microbes
Source: Commun Biol. 2024 Apr 12;7:455. doi: 10.1038/s42003-024-06141-5 (PMC11015016; doi:10.1038/s42003-024-06141-5)

**Supplementary Figures for:**

**Nimble vs. torpid responders to hydration pulse duration among soil microbes**

Patrick Kut^1^ and Ferran Garcia-Pichel^1, 2^

1. Center for Fundamental and Applied Microbiomics and School of Life Sciences, Arizona State University, Tempe AZ, USA
2. Corresponding author. Email: [ferran@asu.edu](mailto:ferran@asu.edu)

**Fig. S1**. Dynamics of oxygenation and soil desiccation in the experimental chambers.**A:** Desiccation dynamics after wetting pulse end based on conductivity 2 mm deep into the soil. An initial period with the vacuum on, and a subsequent desiccation period without vacuum are indicated. Water content of 5%, sufficiently low to halt microbial activity fully, was reached within 4.33 hours. **B**: Desiccation dynamics of a natural crust from the sampling site after a 2.3 mm rain event under summer-like conditions (T=26° C, no wind) shows that desiccation speed can be faster that the one experimentally imposed in A. **C:** Oxygen dynamics at two different depths in the soil during the 60 h treatment incubation. Arrows indicate automated events of DOC and water replenishment in the form of spent *Microcoleus* medium additions. Shaded areas after the second replenishment event are meant to delimit fast (light yellow background) and slow (dark yellow background) soil respiration phases as deduced from the trends in oxygen concentration, indicated progressive consumption and decreasing “quality” of the DOC pool.

**Fig. S2**. Historical yearly rainfall event size distribution at the Jornada LTER (2013-2021); most measurable rain events equal or exceed the wetting experimentally imposed.

**Fig. S3**. Rarefaction Analyses. Curves demonstrate sequencing saturation of all samples at a minimal depth of 11,939 sequences.


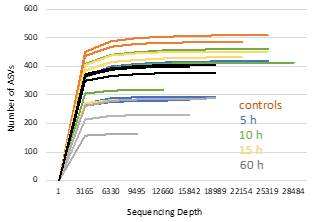


**Fig. S4**. Linear least squares regressions of diversity metrics vs. log of pulse duration, and of microbiome size vs. pulse duration.


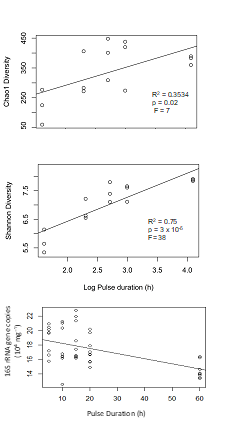


**Fig. S5**. PCoA analyses of microbial community compositional similarity by treatment. Each panel depicts similarity based on different algorithms, according to the legend. Treatments are coded in the same colors across panels, as indicated in the top panel. The percentage of the variability explained by each axis is also indicated. Ellipses, color coded to match the treatments, indicate the 95% confidence area for the respective treatment.


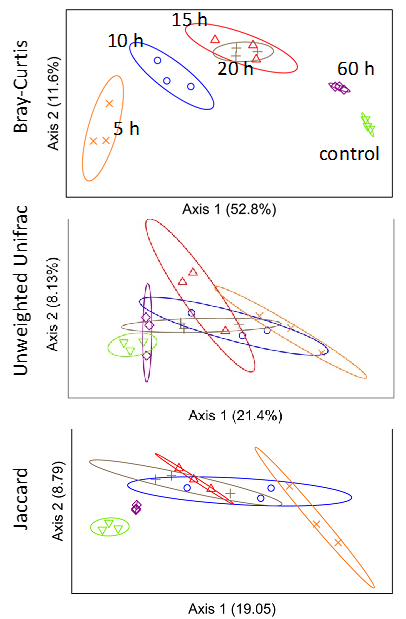


**Fig. S6**. DESeq2- assessed differential ASV abundance (Volcano plot) between the 5 h and 60 h pulse duration treatments. For each ASV, average normalized counts are plotted against their differential abundance (as log base 2-fold change). ASVs that were differentially abundant (p < 0.05) are in red symbols, while non-significant entries are black. Taxonomic assignments are indicated for a few ASVs. Negative values indicate ASV preferring short pulse (NIR) and vice-versa (TOR).


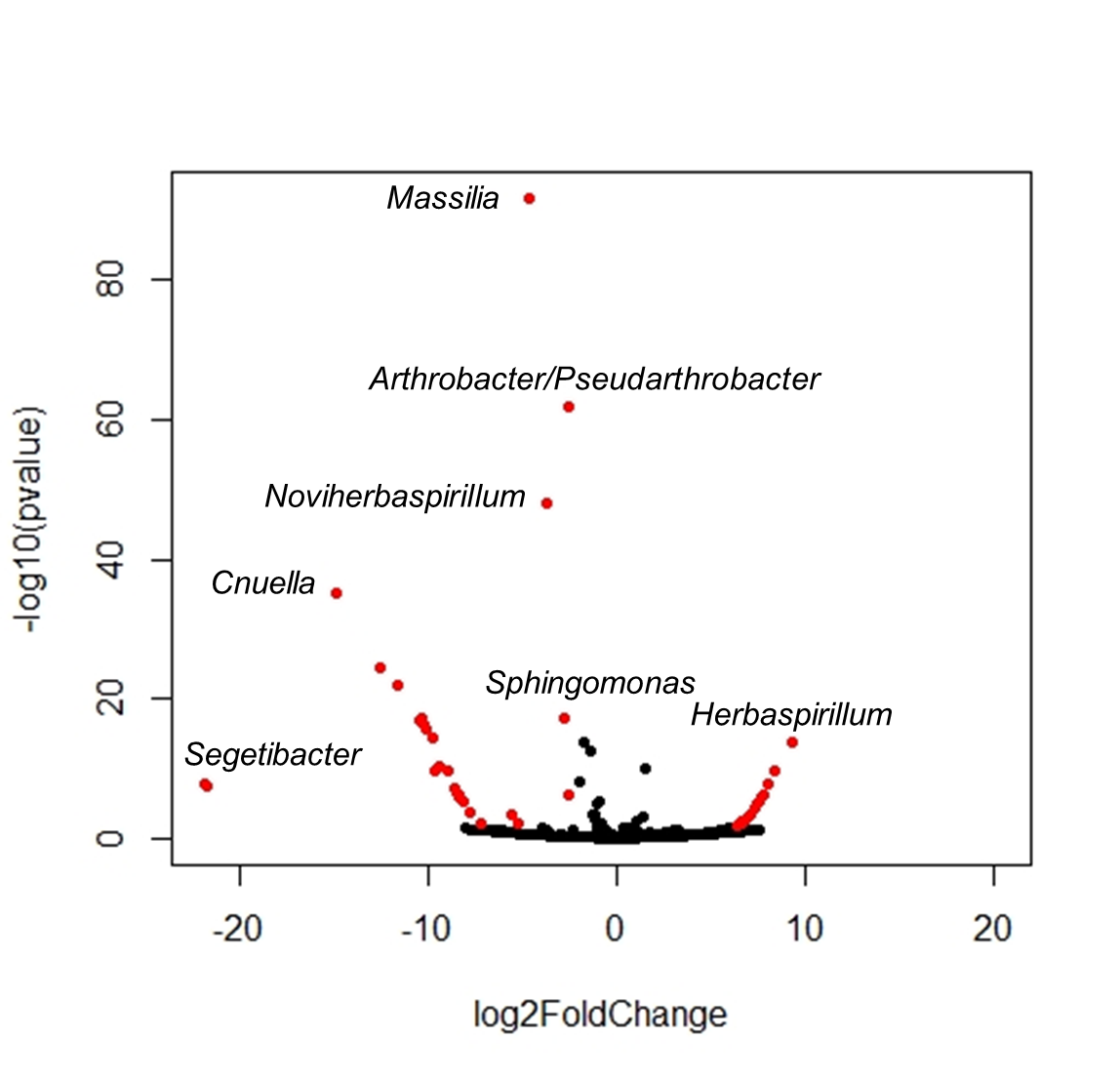

Supplement: Supplementary file 1 — Supplementary Information [file 42003_2024_6141_MOESM1_ESM.docx]
