# Supplementary material for: Nimble vs. torpid responders to hydration pulse duration among soil microbes
Source: Commun Biol. 2024 Apr 12;7:455. doi: 10.1038/s42003-024-06141-5 (PMC11015016; doi:10.1038/s42003-024-06141-5)
Supplement: Supplementary file 2 — Description of Additional Supplementary Files [file 42003_2024_6141_MOESM2_ESM.docx]

**Description of Additional Supplementary Files**

**File name:** Supplementary Data 1
**Description:** Table of ASV specific 16S rRNA gene copies / mg soil determined by multiplying the total 16S rRNA gene copies / mg soil by the proportion of the community composed by each ASV.

**File name:** Supplementary Data 2

**Description:** The p-values associated with the log2-fold change in 16S rRNA gene copies between the 60 h and 5 h treatments for each ASV.

**File name:** Supplementary Data 3

**Description:** The resulting statistical values from linear regression analysis of all ASVs that had at least six data points.

**File name:** Supplementary Data 4

**Description:** Source data behind the graphs in Figure 2.

**File name:** Supplementary Data 5

**Description:** Source data behind the graphs in Figure 4.
